# ‘See’ through the surface: surface-derived three-dimensional AI-driven real-time imaging solution for intra-treatment image guidance

**DOI:** 10.1088/3049-477X/ae3321

**Published:** 2026-01-23

**Authors:** Tingliang Zhuang, Hua-chieh Shao, Ruiqi Li, Tsuicheng Chiu, Kenneth Westover, Steve Jiang, You Zhang

**Affiliations:** 1 Department of Radiation Oncology, University of Texas Southwestern Medical Center, Dallas, TX, United States of America; 2 Medical Artificial Intelligence and Automation Laboratory, University of Texas Southwestern Medical Center, Dallas, TX, United States of America

**Keywords:** real-time 3D imaging, intra-treatment image guidance, motion management, artificial intelligence, surface imaging, dynamic CBCT

## Abstract

Respiratory motion is a long-standing challenge for lung stereotactic body radiotherapy (SBRT), particularly for centrally located lung tumors where increased toxicity demands more precise motion management during treatment. Current two-dimensional imaging approaches are insufficient for 3D tumor deformable motion tracking. In this study, we developed and evaluated a Surface-derived Three-dimensional (3D) AI-driven Real-time (STAR) imaging system by transforming a surface imaging system into a 3D real-time imaging solution. STAR integrated two key components: (1) prior-model-free spatiotemporal implicit neural representation (PMF-STINR): a machine-learning sub-system for pretreatment dynamic cone-beam CT (CBCT) reconstruction and motion modeling; and (2) surface-to-deformation network (Surf2DefNet): a deep-learning model that correlates intra-treatment body surface images with internal 3D anatomy and motion fields, trained based on the dynamic CBCT and motion model output of PMF-STINR. Specifically, PMF-STINR reconstructs a reference CBCT and solves an eigenvector-based motion model from a pretreatment CBCT, while Surf2DefNet predicts the motion eigen-vector weightings from surface images, enabling it to infer real-time CBCTs and motion vector fields (MVFs) using intra-treatment surface maps acquired later. We evaluated STAR imaging using both a digital extended cardiac torso (XCAT) phantom with regular and irregular motion patterns and ten patient datasets. The relative error (RE), center-of-mass error (COME), 95th percentile Hausdorff distance (HD95), Dice coefficients (DICE), and Pearson correlation coefficient (PCC) metrics between STAR images and the ‘GT’ were evaluated. For the XCAT phantom and the ten patients, the mean COME values are within 1 mm for all but one patient (1.3 mm). The RE values were consistently low, and the DICE and PCC values exceeded 0.89 in all cases. The HD95 are all within 2 mm except for one patient (2. 78 mm). These results demonstrate that the STAR imaging system can achieve accurate spatiotemporal reconstructions from surface images, providing CBCTs and MVFs for intra-treatment real-time image-guidance, and has the potential to improve safety and efficacy of SBRT for lung cancer.

## 1. Introduction

The advent of image-guided radiotherapy, using on-board x-ray cone-beam CT (CBCT) or other in-room imaging systems, has transformed cancer treatment. The patient’s internal anatomy, ‘seen’ through the x-ray system, provides three-dimensional (3D) geometrical information for accurate tumor targeting [1] and, more recently, for online adaptive treatments to account for anatomical changes [2]. While optimal patient setup guided by pretreatment imaging may be achieved before radiation delivery, intra-treatment motion of the tumor and organ brings significant challenges to maintaining precise targeting. Accurate tumor targeting during treatment is critical for stereotactic body radiotherapy (SBRT), where a large dose of radiation is delivered to small tumors in ⩽5 treatment fractions with sharp spatial dose gradients. Geometric misses can result in severe tumor underdosing and increased normal tissue toxicity.

Currently, moving tumors can be treated using the free-breathing strategy, which employs a motion encompassing approach by designing an internal target volume (ITV) from a four-dimensional CT (4D CT). The 4D CT is used to characterize the target motion range. An additional margin is added to the ITV to generate the planning target volume, accounting for additional positioning/motion uncertainties. A sufficiently large margin can avoid geometric misses, but inevitably increases the irradiation of normal tissues, leading to higher radiation toxicity [3]. Motion management techniques, including breath-hold and respiratory gating, have been implemented to confine radiation to a predefined tumor motion window to reduce irradiation volumes [4, 5] and the associated toxicities. Such techniques, however, require real-time monitoring and tracking of tumor motion. Currently, they often rely on measurable external motion (usually via a single point-based surrogate) to represent internal tumor motion, which is error-prone [6–9]. An ideal solution for intra-treatment motion monitoring/management would be real-time CBCT imaging to allow direct tumor visualization and volumetric anatomy tracking. However, current clinical imaging systems for real-time tumor monitoring are mostly limited to two-dimensional (2D) approaches [10], which are inadequate to track tumors’ 3D deformable motion. Planar radiographic imaging using implanted markers as surrogates has been used for tumor localization during radiotherapy delivery. This invasive procedure, however, is rare for the treatment of centrally located lung tumors due to the risks of pneumothorax [11]. The emerging MRI linear accelerator provides the potential to visualize patient anatomy during treatment without additional radiation dose. However, besides high cost and low availability, current technology only allows imaging of a 2D slice at a time, lacking motion information in all dimensions. The widely available on-board CBCT imaging has been used to provide 3D imaging for initial patient setup. However, it cannot provide real-time intra-treatment imaging due to its long scan time (∼1 min). Real-time 3D imaging for intra-treatment image guidance is yet to be developed.

Advancements in artificial intelligence (AI) have revolutionized our ability to model complex nonlinear relationships, enabling innovative solutions to medical imaging challenges [12, 13]. Many studies have demonstrated direct reconstruction of 3D CBCT images from extremely limited x-ray projections via deep-learning models [14–28]. The robustness of such models, however, remains to be validated and improved in real clinical scenarios, as direct 2D-to-3D intensity mapping can be highly unstable. Another approach is to build a motion model using prior patient information (such as 4D CT) and infer real-time 3D CBCT during treatment by using an x-ray projection [29], or a surrogate signal such as surface images [30–35]. The surface image-based approach is inherently compatible with rotating gantry systems due to the fixed camera for image acquisition. It does not incur additional radiation dose, either. However, these studies used surface deformation [30] or whole body surface image [32–34] as the network input, which are not directly measurable in the current surface imaging systems. In addition, the accuracy of current x-ray-based and surface image-based approaches is limited by a common factor: they rely on a planning 4D CT for model training. Residual motion artifacts due to non-periodic or irregular motion in 4D CT not only degraded the image quality but also introduced errors in motion modeling. Furthermore, it is challenging for a 4D CT-based motion model to account for variations in the relationship between surrogate signals and internal motion that occur from the time of planning 4D CT acquisition to treatment delivery. Pretreatment 4D CBCT could be used to build an updated motion model. However, current 4D CBCT similarly suffers from artifacts due to breathing motion irregularity and projection undersampling. These residual artifacts can propagate to the generated images and thus negatively impact the motion tracking accuracy.

Recently, Shao *et al* developed a deep learning based technique: prior-model-free spatiotemporal implicit neural representation (PMF-STINR) to reconstruct motion artifact-free dynamic CBCTs from a standard one-minute data acquisition [36]. Unlike conventional 4D CBCT imaging, PMF-STINR does not require phase sorting/binning and can resolve both regular and irregular motion by reconstructing a CBCT volume from each individual x-ray projection. It can faithfully capture the most up-to-date, motion artifact-free patient anatomy and provide a consistent motion model for estimating its spatiotemporal deformation before each treatment session. Building on previous work modeling the complex non-linear relationship between the surface and internal motion, we developed a deep-learning model that links the real-time surface images to the temporal components of the PMF-STINR-based internal anatomy motion/deformation model. By combining the surface derived temporal information with spatial motion information from the PMF-STINR framework, real-time 3D CBCTs can be generated. In this study, we propose the Surface-derived 3D AI-powered Real-time (STAR) imaging system, which integrates a surface-to-deformation model with the PMF-STINR dynamic CBCT framework, to address the critical need for real-time intra-treatment image guidance in motion management. We hypothesize that the STAR imaging system can generate high-fidelity, real-time 3D images for intra-treatment guidance, and tested this hypothesis using both digital phantom and patient data in this study.

## 2. Methods

### 2.1. Overview

**Figure 1.**
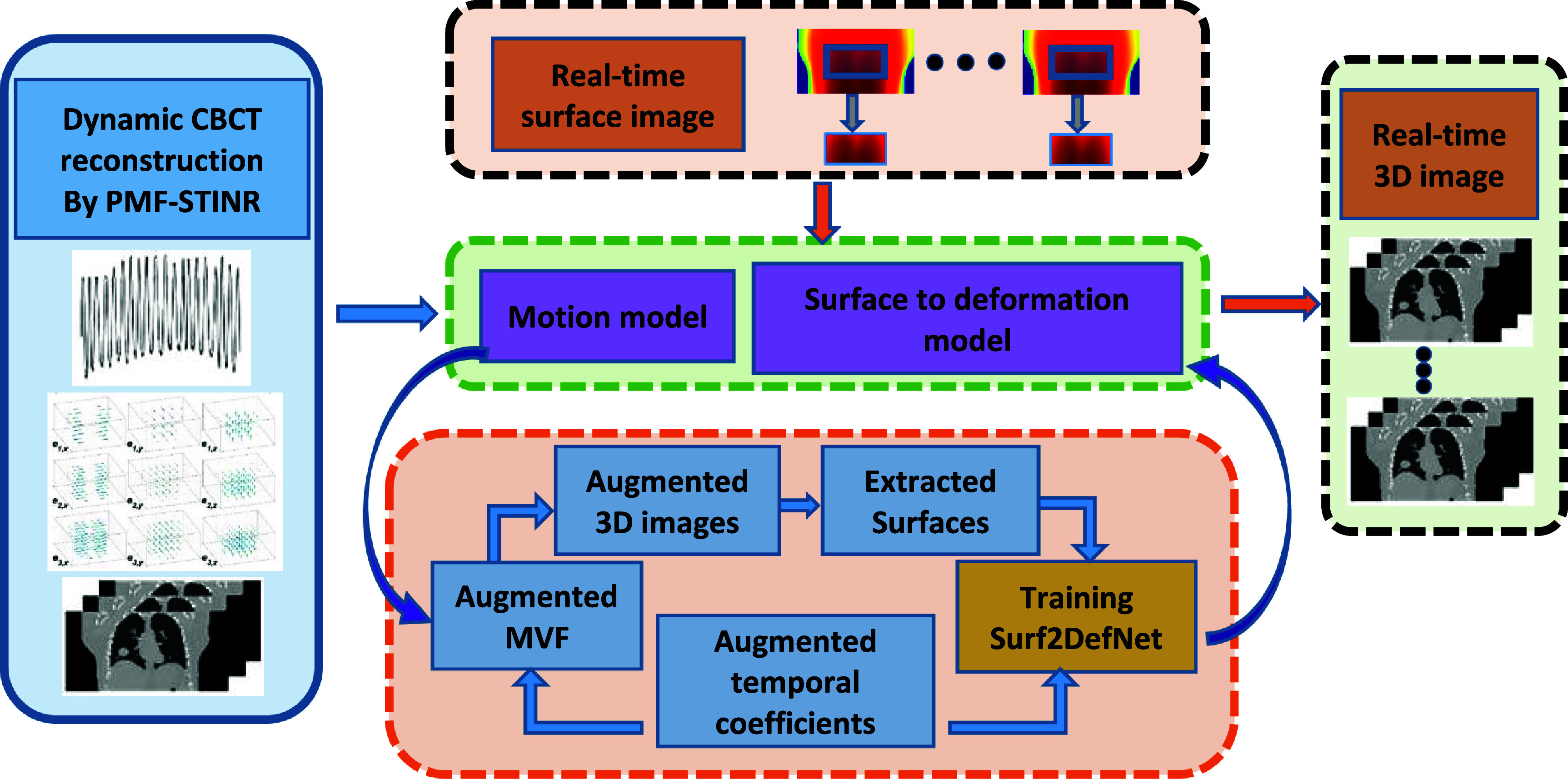
Overview of the STAR imaging.

As shown in figure 1, the two key algorithms of STAR are (1) PMF-STINR for dynamic CBCT reconstruction and motion model building; and (2) CNN-based surface-to-deformation network (Surf2DefNet) for surface imaging to real-time CBCT mapping and motion tracking.

#### 2.1.1. Personalized motion model based on dynamic CBCT reconstruction

The pretreatment dynamic CBCT reconstruction by PMF-STINR will result in a time series of 3D images denoted by ${\mathrm{CBC}}{{\mathrm{T}}_{{\mathrm{dyn}}}}\left( t \right)$. The details of this algorithm can be found in [36]. In brief, by decoupling image reconstruction and motion estimation, the problem of reconstructing 3D images at a given time ${\text{ }}t$ in the presence of motion is turned into solving a reference 3D image ${\mathrm{CBC}}{{\mathrm{T}}_{{\mathrm{ref}}}}$, and the motion vector field (MVF) ${\boldsymbol{D}}\left( t \right)$ between ${\mathrm{CBC}}{{\mathrm{T}}_{{\mathrm{ref}}}}$ and ${\mathrm{CBC}}{{\mathrm{T}}_{{\mathrm{dyn}}}}\left( t \right)$, which are related by the following equation:

\begin{equation*}{\text{ CBC}}{{\mathrm{T}}_{{\mathrm{dyn}}}}\left( t \right) = {\mathrm{CBC}}{{\mathrm{T}}_{{\mathrm{ref}}}}\left( {\boldsymbol{x}} + {\boldsymbol{D}}\left( t \right) \right){\text{ }}.\end{equation*}

To leverage the redundancy of motion fields for dimension reduction, the MVF is decomposed into spatial and temporal components as follows:

\begin{equation*}{\boldsymbol{D}}\left( t \right) = {\text{ }}\sum\limits_{i = 1}^3 {{\boldsymbol{a}_i}} \left( t \right){\boldsymbol{e}_i},\end{equation*}

where ${\boldsymbol{e}_i}$ are three ($i = 1,2,3$) motion basis components (eigen-modes) of the motion fields, with ${\boldsymbol{a}_i}\left( t \right)$ the corresponding temporal coefficients at a given time point $t$. Equations (1) and (2) form the basis for a personalized motion model, allowing the generation of a 3D image at a later time $t$ (post dynamic CBCT acquisition) by determining new temporal coefficients ${\boldsymbol{a}_i}\left( t \right)$ based on new intra-treatment imaging signals, i.e. surface imaging maps for STAR.

#### 2.1.2. Surface to deformation prediction model: Surf2DefNet

Based on the motion model (${\boldsymbol{e}_i}$) and the reference CBCT (${\mathrm{CBC}}{{\mathrm{T}}_{{\mathrm{ref}}}}$) output by PMF-STINR, a CNN-based Surf2DefNet will map intra-treatment surface images into 3D CBCT volumes for real-time motion tracking. Specifically, input to Surf2DefNet will be a surface image within a preselected region-of-interest (surfROI) acquired at time $t$ during the treatment. The pixel-wise height of this surface image, in relation to a reference surface extracted from ${\mathrm{CBC}}{{\mathrm{T}}_{{\mathrm{ref}}}}$, will be fed into a CNN to yield the temporal coefficients ${\boldsymbol{a}_i}\left( t \right){\text{ }}$ (see equation (2)). Combined with the motion model ${\boldsymbol{e}_i}$, the derived temporal coefficients will generate a real-time MVF ${\boldsymbol{D}}\left( t \right)$, which describes the real-time volumetric motion and maps ${\mathrm{CBC}}{{\mathrm{T}}_{{\mathrm{ref}}}}$ into an intra-treatment 3D image ${\mathrm{CBC}}{{\mathrm{T}}_{{\mathrm{STAR}}}}$.

**Figure 2.**
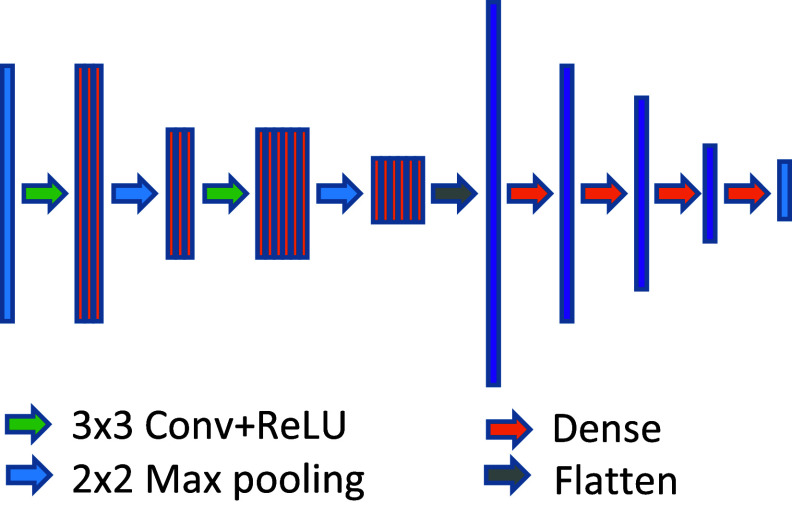
The convolutional neural network architecture of Surf2DefNet.

The architecture of the CNN model is shown in figure 2. The Surf2DefNet consists of two 3 × 3 convolution layers with ReLU activation, each followed by a 2 × 2 max-pooling layer. The number of filters for the first and the second convolution layers is 32 and 64, respectively. The output from the second max-pooling layer is flattened and passed through three fully connected layers with 128, 64, and 32 units, all with ReLU activation. The mean squared error (MSE) between the derived and the ‘ground-truth’ (GT) temporal coefficients from the training data is minimized in the CNN model.

### 2.2. Data

We used both the extended cardiac torso (XCAT) digital phantom [37] and real patient data to evaluate STAR imaging.

#### 2.2.1. XCAT phantom

XCAT phantom is a highly detailed computational model of the human anatomy that includes respiratory motion modeling. We simulated a regular (*X*1) and irregular (*X*2) breathing motion with XCAT in this study. The dynamic CBCT reconstruction was performed by PMF-STINR, on projection data simulated by the tomographic ASTRA toolbox [38]. A total of 660 projections were simulated in a one-minute scan as in the data acquisition on a clinical CBCT system with a full-fan mode. The images were reconstructed with a matrix size of 200 × 200 × 100 and an isotropic resolution of 2 mm. To avoid lateral data truncation, we used a virtual detector with 400 × 192 elements with pixel size of 1.55 mm^2^ to cover the entire phantom.

#### 2.2.2. Patient cases

Data from 10 patients (P1–P10) were used to evaluate the STAR imaging. Among these patient datasets, P1 is from MD Anderson Cancer Center, P3–4 from the SPARE challenge dataset [39], and P2 and P5–P10 from UT Southwestern Medical Center. Both full-fan and half-fan CBCT dataset were included in this study. The dynamic CBCTs were reconstructed with a matrix size of 200 × 200 × 100 in full-fan acquisition and 300 × 300 × 102 in half-fan mode, using an isotropic voxel size of 2 mm for all patients except P5. For patient P5, the reconstruction matrix size was 310 × 310 × 102. The detailed data acquisition parameters can be found in the appendix.

Due to the large number of dynamic CBCTs reconstructed by PMF-STINR (one CBCT for each x-ray projection), we downsampled each set of dense dynamic CBCT images to remove redundant information before training and testing in patient studies. For patients with more than 2000 projections due to the slow gantry rotation acquisition, a downsampling factor of 4–5 was used to extract a subset from the dynamic CBCT sequence. For patients with 895/894 projections, every third CBCT was discarded, resulting in a downsampling factor of 1.5. For patient P2, the downsampling factor was 2. The motion curves in the Results section represent the patients’ breathing patterns sampled at these discretized time points.

**Figure 3.**
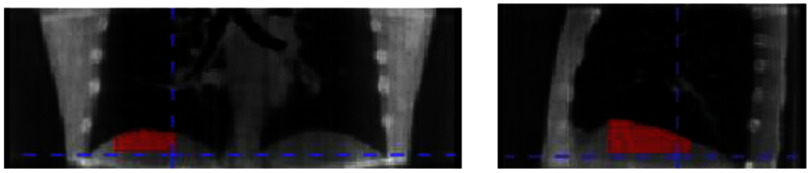
An example of the diaROI.

To quantify the motion tracking accuracy, we created a region-of-interest including the diaphragm region-of-interest (labeled as diaROI) as a motion surrogate for evaluation. For scans that did not include the diaphragm, a visible lung nodule/feature was used as the motion surrogate. Figure 3 shows the delineation of the diaROI for one of the patients.

### 2.3. Implementation

#### 2.3.1. Surface image

**Figure 4.**
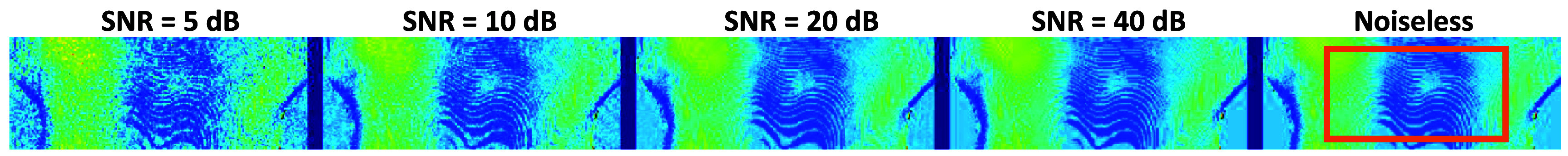
Surface images with different levels of Gaussian noise. A manually selected rectangle surfROI was demonstrated on the noiseless surface image.

In this study, surface images were simulated using the boundary voxels of the dynamic CBCTs based on a manually selected threshold for edge detection. At each axial slice, the relative surface height was defined as the pixel-wise difference in the anterior-posterior coordinates of the boundary pixels between the dynamic CBCT at time $t$ (${\mathrm{CBC}}{{\mathrm{T}}_{{\mathrm{dyn}}}}\left( t \right)$) and the reference CBCT (${\mathrm{CBC}}{{\mathrm{T}}_{{\mathrm{ref}}}}$). In addition, to simulate the impact of imperfect surface data acquisition with a camera system on the prediction accuracy of Surf2DefNet, Gaussian white noise with different signal-to-noise ratios (SNR = 5, 10, 20, 40 dB) was introduced on the relative surface height. Note that the ‘signal’ corresponds to the relative surface change due to breathing, which represents the surface contrast. An example of surface images with different levels of noise is shown in figure 4.

As described in the overview, the relative surface height within a preselected surfROI was fed into the Surf2DefNet. A rectangular surfROI (figure 4) was manually determined for each case in this feasibility study. However, the shape and size determination of the surfROI can be potentially automated and optimized.

#### 2.3.2. Training of the Surf2DefNet

To train the CNN model to infer temporal coefficients from surface map inputs, a large amount of high-quality training data augmented from the dynamic CBCT set was used. Augmentation helps to increase the training sample size and create new motion outside of the original dynamic CBCT set to train a more robust CNN model. Specifically, by applying random scaling factors ${\lambda _i}$ to the temporary coefficients at selected time points $t \in {T_0}$, different motion states can be created.

\begin{equation*}\tilde {\boldsymbol{D}}\left( t \right) = \sum\nolimits_{i = 1}^3 {{{\tilde {\boldsymbol{a}_i}}}\left( t \right){\boldsymbol{e}_i},} {\text{ where }}{\tilde {\boldsymbol{a}}_i}\left( t \right) = {\text{ }}{\lambda _i}{\tilde {\boldsymbol{a}}_i}\left( t \right),{\text{ }}t \in {T_0}\end{equation*}

where ${\lambda _i}$ were randomly sampled from a uniform distribution. Deforming ${\mathrm{CBC}}{{\mathrm{T}}_{{\mathrm{ref}}}}$ by $\tilde {\boldsymbol{D}}\left( t \right)$ will generate augmented CBCTs (equation (1)), from which surface maps can be extracted. Using these surface maps with known temporal coefficients ${\widetilde {\boldsymbol{a}}_i}\left( t \right)$, Surf2DefNet can be trained in a supervised fashion to infer motion coefficients from intra-treatment surface images. The loss function was the MSE between the output and the ‘GT’ temporal coefficients $\tilde a_i^k\left( t \right)$, where $k$ denotes the three Cartesian directions *x, y*, and *z*. Three separate trainings were performed for the three Cartesian components of the MVF.

We manually set ${T_0}$ (equation (3)) to contain 60–80 equally spaced time points, covering about 80% of total time points in the original dynamic CBCT series. The number of random samples of ${\lambda _i}$ in the ranges of [0.5, 2] for $i = 1,2,3$ was set to be 3, 3, and 2, respectively. As an example, for original dynamic CBCT series containing 596 CBCTs, 80 were sampled from the 596*80% = 480 CBCTs to generate 80 × 3 × 3 × 2 = 1440 augmented CBCTs for training. Including the 480 original CBCTs, the total number of training samples was 1440 + 480 = 1920, which was randomly partitioned into 80% for model training and 20% for validation. The final model was selected based on the lowest validation loss observed during training. An NVIDIA GeForce RTX 4070 Ti SUPER GPU with 16GB memory was used to train this model.

### 2.4. Evaluation

For the digital phantom study, we used the phantom image and programmed motion as the ‘GT’ to assess the performance of STAR imaging. For patient study, because there is no ‘GT’ images, we will use the dynamic CBCT images that were not ‘seen’ by the Surf2DefNet model, as references to evaluate the STAR images.

The following common metrics were used to quantify the imaging performance. To quantify the spatial-temporal anatomy tracking accuracy, we calculated the tumor/diaROI center-of-mass (COM) and COM error (COME =$|{\overset{{\scriptscriptstyle\rightharpoonup}} {r} _{{\mathrm{STAR}}}}-$${\overset{{\scriptscriptstyle\rightharpoonup}} {r} _{{\mathrm{GT}}}}|$), which represents the Euclidean distance between the COM on the STAR images ${\overset{{\scriptscriptstyle\rightharpoonup}} {r} _{{\mathrm{STAR}}}}$ to the ‘GT’ ${\overset{{\scriptscriptstyle\rightharpoonup}} {r} _{{\mathrm{GT}}}}$. The tumor/diaROI shape similarity is quantified by the Dice coefficients (DICE = $2{V_{{\mathrm{STAR}}}} \cap {V_{{\mathrm{GT}}}}/{V_{{\mathrm{STAR}}}} \cup {V_{{\mathrm{GT}}}}$) and 95th percentile Hausdorff distance (HD95) between the contours obtained on the two image sets. These contours were initially delineated manually on the reference CBCT images and subsequently propagated to the 3D images at other time points using inferred MVFs. In addition, the similarity of two motion profiles was quantified by the Pearson correlation coefficient (PCC). For overall spatial accuracy evaluation, we used the relative error (RE) metric to quantify the voxel-wise differences, defined as follows:

\begin{equation*}RE = {\text{ }}\frac{1}{{{N_t}}}\mathop {\mathop \sum \nolimits }\limits_t \sqrt {\frac{{{{\mathop \sum \nolimits }_{\overset{{\scriptscriptstyle\rightharpoonup}} {x} }}{{\left| {\left| {{I^{{\mathrm{STAR}}}}\left( {\overset{{\scriptscriptstyle\rightharpoonup}} {x} ,t} \right) - {I^{{\mathrm{GT}}}}\left( {\overset{{\scriptscriptstyle\rightharpoonup}} {x} ,t} \right)} \right|} \right|}^2}}}{{{{\mathop \sum \nolimits }_{\overset{{\scriptscriptstyle\rightharpoonup}} {x} }}{{\left| {\left| {{I^{{\mathrm{GT}}}}\left( {\overset{{\scriptscriptstyle\rightharpoonup}} {x} ,t} \right)} \right|} \right|}^2}}}} \end{equation*}

where ${I^{{\mathrm{STAR/GT}}}}\left( {\overset{{\scriptscriptstyle\rightharpoonup}} {x} ,t} \right)$ is the pixel value at $\overset{{\scriptscriptstyle\rightharpoonup}} {x} $ for the STAR/GT 3D images at the time point $t$.

## 3. Results

### 3.1. The XCAT study

**Figure 5.**
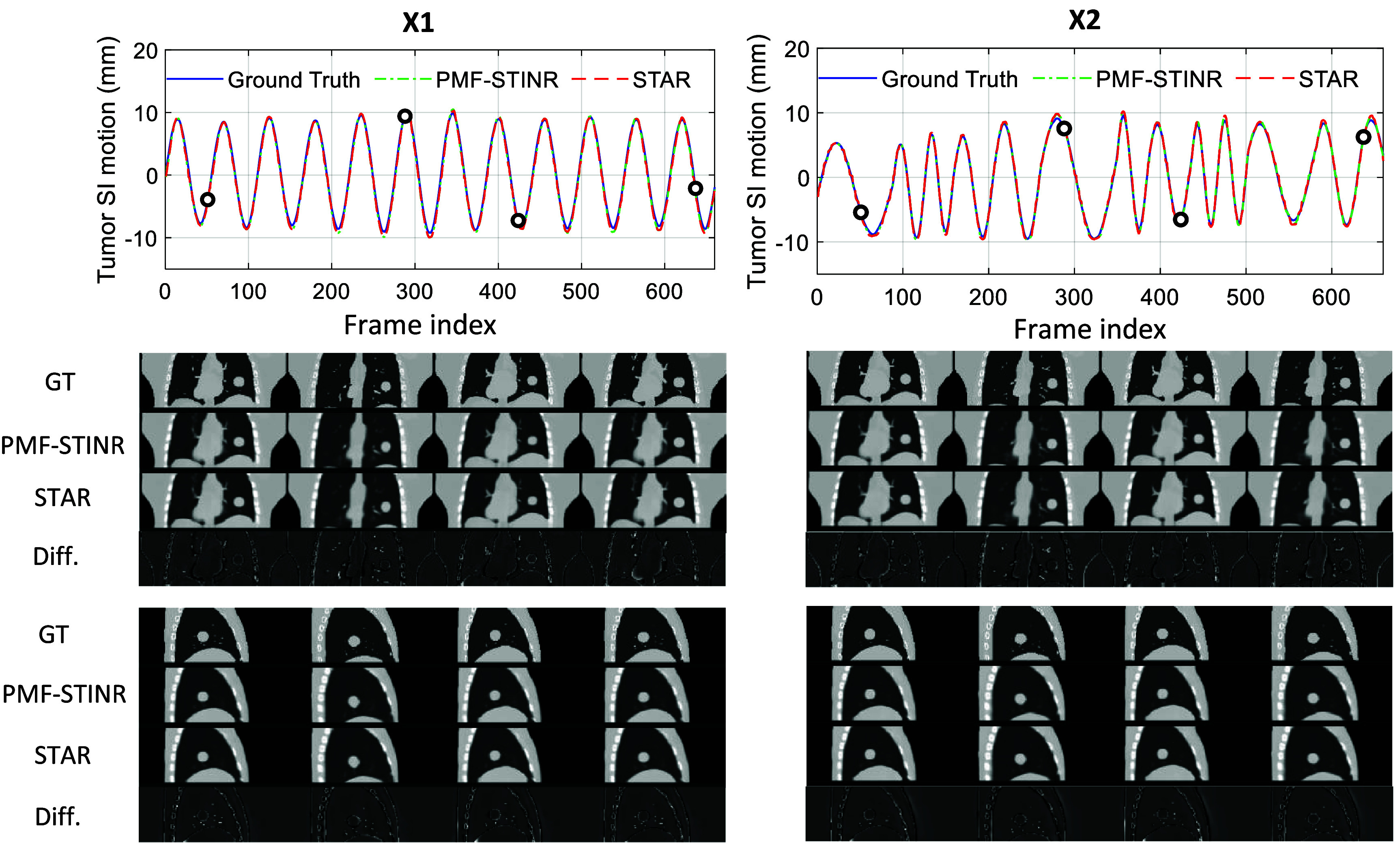
Motion profile and sectional image comparisons of the XCAT study for motion scenarios X1 (left) and X2 (right). The top panel shows the motion profiles in the SI direction. The second and third panels show the coronal and sagittal images at four selected time indices, as marked by the black circles on the motion profiles. The fourth row of these two panels shows the difference images between the STAR CBCT and the phantom ‘ground truth’. The sectional images are displayed in the window [−1000, 210] HU and the difference in the window [−1200, −270] HU.

Figure 5 shows comparisons of tumor motion profiles and cross-sectional images at multiple time points between the phantom ‘GT’, the dynamic CBCT via PMF-STINR, and the STAR CBCT for both regular (*X*1) and irregular (*X*2) motion scenarios. Simulated surface images without noise were used as inputs to Surf2DefNet. The first panel shows excellent agreement in motion profiles along the SI direction. Coronal and sagittal slices are shown at four representative time points, indicated by the black circles on the motion curves. Difference images between the GT and the STAR CBCTs, shown in the fourth row of both the middle and the bottom panels, further illustrate good spatial agreement.

**Figure 6.**
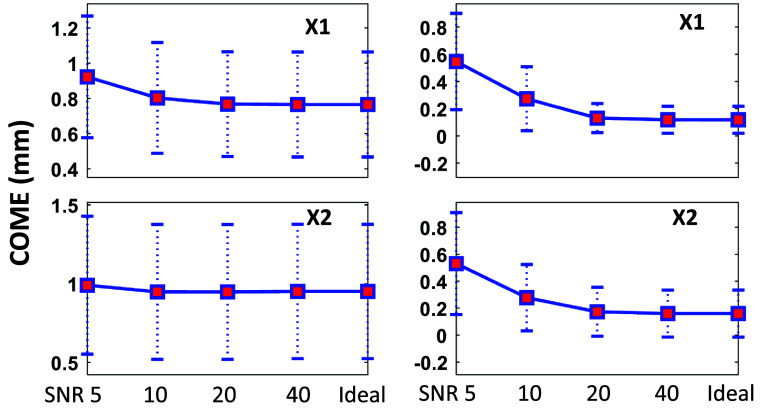
Tumor center-of-mass error (COME) of STAR CBCTs with varying levels of Gaussian noise added to the surface image input, relative to the phantom ‘ground truth’ (left) and the dynamic CBCTs (right). Upper row: motion scenario X1; lower row: scenario X2.

Figure 6 shows the tumor localization accuracy (COME) under varying levels of Gaussian noise added to the simulated surface images for the two motion scenarios. As noise levels decreased, the mean localization accuracy improved when comparing STAR CBCT images to both the phantom ‘GT’ and the dynamic CBCTs. Table 1 summarizes quantitative comparisons of STAR CBCT images generated from both ideal and noisy surface inputs (SNR = 5 dB) to (a) the phantom ‘GT’ and (b) the dynamic CBCTs, using four metrics: COME, DICE, RE, and PCC. Across all test cases, the mean localization error remained below 1 mm, the DICE were greater than 0.9, and the PCC values were near unity. The DICE, RE, and PCC demonstrated minimal sensitivity to the presence of noise.

**Table 1.** Accuracy of STAR imaging in the XCAT phantom study, in terms of tumor COME and DICE, relative error (RE), and Pearson correlation coefficient (PCC) with noiseless and noisy surface image input.

**(a) Comparison between the STAR CBCTs and the phantom ‘ground truth’**								

	**COME(mm)**		**DICE**		**RE**		**PCC**	
	**Noiseless**	**SNR 5 dB**	**Noiseless**	**SNR 5 dB**	**Noiseless**	**SNR 5 dB**	**Noiseless**	**SNR 5 dB**
*X*1	0.77 ± 0.30	0.92 ± 0.35	0.884 ± 0.012	0.881 ± 0.012	0.147 ± 0.006	0.147 ± 0.005	0.999	0.999
*X*2	0.95 ± 0.43	0.99 ± 0.44	0.903 ± 0.016	0.902 ± 0.015	0.151 ± 0.009	0.151 ± 0.009	0.999	0.999

**(b) Comparison between the STAR CBCTs and the dynamic CBCTs (based on PMF-STINR).**								

	**COME(mm)**		**DICE**		**RE**		**PCC**	
	**Noiseless**	**SNR 5 dB**	**Noiseless**	**SNR 5 dB**	**Noiseless**	**SNR 5 dB**	**Noiseless**	**SNR 5 dB**

*X*1	0.11 ± 0.10	0.55 ± 0.35	0.995 ± 0.005	0.975 ± 0.015	0.005 ± 0.002	0.018 ± 0.004	1.000	0.999
*X*2	0.16 ± 0.17	0.53 ± 0.38	0.993 ± 0.007	0.976 ± 0.016	0.006 ± 0.004	0.016 ± 0.003	1.000	0.998

Larger differences, particularly in the RE metric, were observed when comparing STAR images to the phantom ‘GT’ than the dynamic CBCTs, as shown in table 1. This is due to the contribution of additional uncertainties in the dynamic CBCT reconstruction, which STAR is based on, to voxel-level image differences. However, they are comparable to those in [36].

### 3.2. The patient study

For the patient study, where ‘GT’ CBCTs are not available, dynamic CBCT images excluded from Surf2DefNet training were used as the reference data to evaluate the STAR imaging performance.

**Figure 7.**
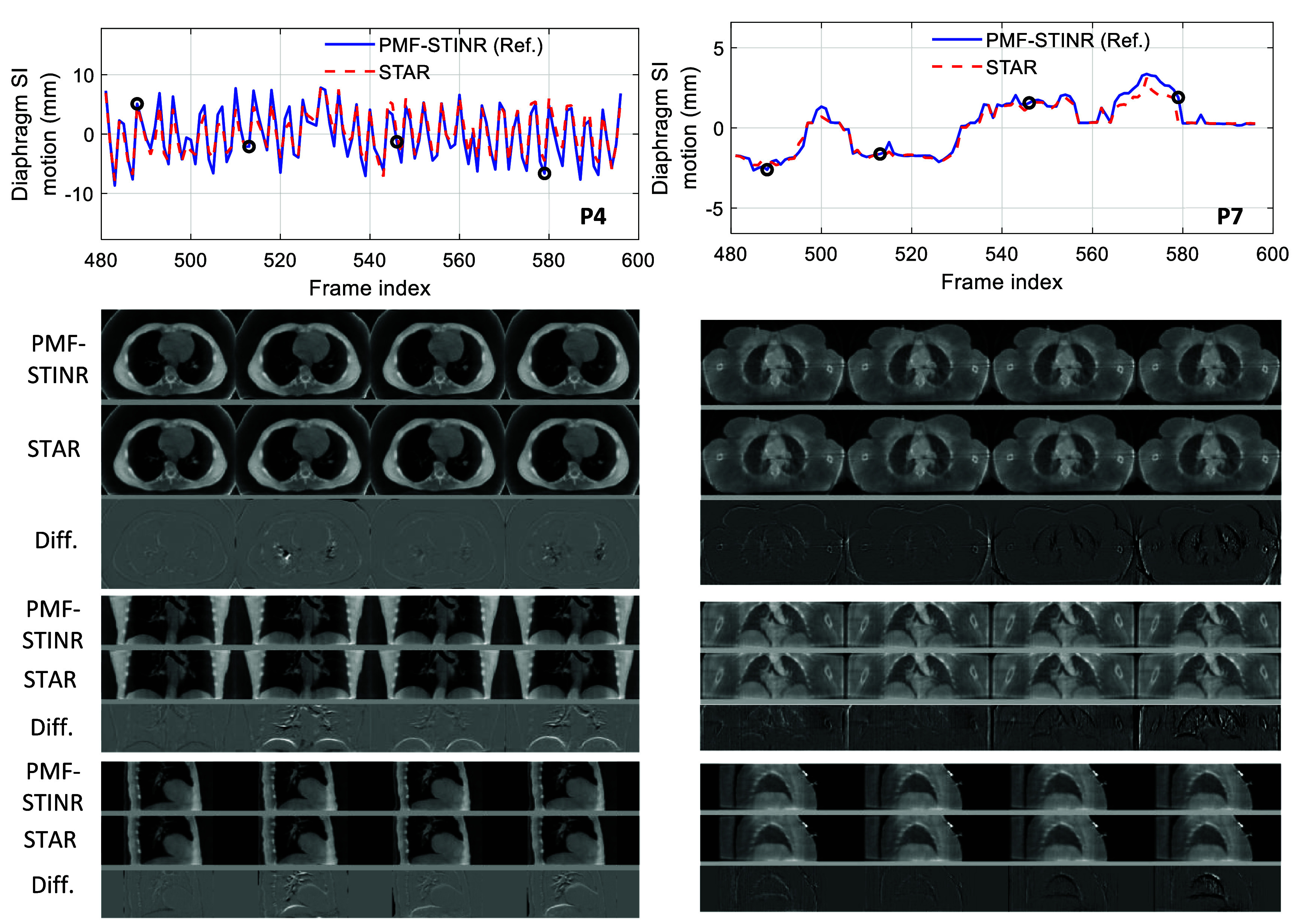
Motion profile and sectional image comparisons of the patient study for two example patients: P4 (left) and P7 (right). The top panel shows the diaphragm motion profiles in the SI direction. The second, third, and fourth panels show the axial, coronal, and sagittal images at four selected time indices, marked by the black circle on the motion profiles. The third row of these three panels shows the difference images between the STAR CBCTs and the dynamic CBCTs (based on PMF-STINR). The display window for P4 is [−1000, 820] HU for the sectional images and [−1360, −640] HU for the difference images. The display window for P7 is [−1000, −170] HU for the sectional images and [−1020, −950] HU for the difference images.

Figure 7 shows comparisons of images and motion tracking results between the STAR CBCTs and the dynamic CBCTs for two representative patients. Surface images were simulated from the patients’ body outline without adding noise. Using the COM of the diaROI as a motion surrogate, the first panel demonstrated strong agreement between the diaphragm motion profiles derived from STAR images and the dynamic CBCT references. The corresponding coronal and sagittal slices at the four selected time points reveal minimal anatomical discrepancies, confirming the overall high spatial fidelity of the STAR imaging. Note that the apparent high frequency variation in the motion profile of P4 as compared to P7 does not reflect the actual temporal breathing pattern, since the abscissa represents a dimensionless time index as mentioned in section 2.2.

**Figure 8.**
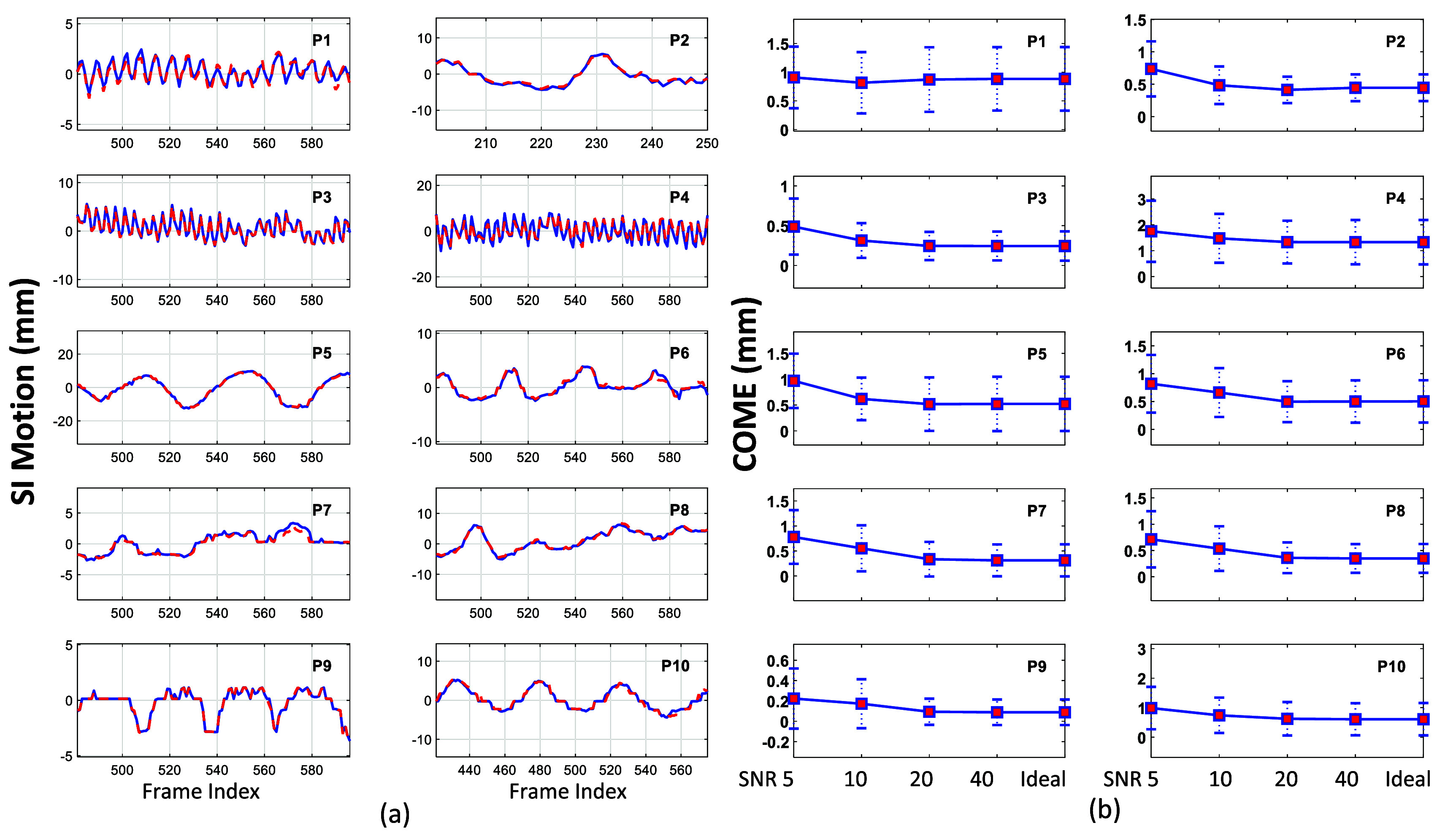
(a) Diaphragm/lung feature motion profile comparisons in the SI direction for all patients. The red dashed curves represent motion extracted from the STAR CBCTs, and the blue solid curves correspond to motion from the dynamic CBCTs. (b) Error bar plots of the diaphragm/lung feature ROI center-of-mass error of STAR CBCTs relative to the dynamic CBCTs, with varying levels of Gaussian noise added to the surface image input, for all patients.

Figure 8(a) shows the diaphragm/lung feature motion profiles in the SI direction for all patient cases, while figure 8(b) presents the localization accuracy with varying levels of Gaussian noise added to the simulated surface images. As seen in the motion profile comparisons, good agreement was achieved across different breathing patterns, for instance, in P8, which exhibited a significant baseline shift. Quantitative metrics, including COME, DICE, RE, and PCC, are summarized in table 2. Except for P4, the COME values were below 1 mm and HD 95 were below 2 mm (one pixel) for both noise-free and noisy surface input. Similar to the phantom study, localization accuracy improved with reduced noise. For all cases, the localization errors were within 2 mm, and the PCC values exceeded 0.9, demonstrating accurate anatomy tracking. These results confirm the robust performance of STAR imaging in motion tracking and its ability to generate high-fidelity, real-time 3D images across a wide range of breathing irregularities.

**Table 2.** Accuracy of STAR imaging compared to the dynamic CBCTs in the patient study, in terms of diaphragm/lung feature ROI COME and DICE, relative error (RE), and Pearson correlation coefficient (PCC) with noiseless and noisy surface image input.

Patient ID	COME (mm)		HD95 (mm)		DICE		RE		PCC	
	No noise	SNR 5 dB	No noise	SNR 5 dB	No noise	SNR 5 dB	No noise	SNR 5 dB	No noise	SNR 5 dB
P1	0.88 ± 0.55	0.91 ± 0.54	2.00 ± 0.23	1.97 ± 0.34	0.893 ± 0.062	0.888 ± 0.062	0.032 ± 0.012	0.035 ± 0.011	0.913	0.876
P2	0.45 ± 0.21	0.74 ± 0.43	1.76 ± 0.65	1.95 ± 0.43	0.972 ± 0.016	0.947 ± 0.032	0.013 ± 0.006	0.019 ± 0.007	0.994	0.973
P3	0.24 ± 0.19	0.49 ± 0.35	1.48 ± 0.88	1.93 ± 0.37	0.982 ± 0.013	0.963 ± 0.023	0.014 ± 0.007	0.026 ± 0.007	0.993	0.973
P4	1.33 ± 0.86	1.75 ± 1.19	2.78 ± 1.14	4.07 ± 2.68	0.938 ± 0.039	0.911 ± 0.057	0.059 ± 0.014	0.066 ± 0.012	0.953	0.917
P5	0.52 ± 0.53	0.97 ± 0.53	1.41 ± 1.00	2.12 ± 0.45	0.971 ± 0.032	0.925 ± 0.046	0.008 ± 0.006	0.024 ± 0.007	0.998	0.992
P6	0.50 ± 0.38	0.82 ± 0.52	1.45 ± 0.90	1.87 ± 0.62	0.977 ± 0.019	0.959 ± 0.031	0.044 ± 0.020	0.049 ± 0.018	0.980	0.929
P7	0.31 ± 0.32	0.78 ± 0.54	1.16 ± 0.99	1.57 ± 0.83	0.983 ± 0.022	0.955 ± 0.037	0.023 ± 0.014	0.031 ± 0.013	0.984	0.920
P8	0.35 ± 0.27	0.71 ± 0.54	1.17 ± 0.99	1.62 ± 0.79	0.979 ± 0.020	0.953 ± 0.036	0.043 ± 0.033	0.048 ± 0.031	0.993	0.977
P9	0.09 ± 0.13	0.22 ± 0.30	0.78 ± 0.99	1.45 ± 0.91	0.992 ± 0.011	0.980 ± 0.026	0.010 ± 0.006	0.014 ± 0.005	0.995	0.968
P10	0.61 ± 0.55	0.99 ± 0.72	0.78 ± 0.99	1.81 ± 0.74	0.959 ± 0.011	0.917 ± 0.047	0.004 ± 0.006	0.015 ± 0.005	0.980	0.952

### 3.3. Robustness evaluation of STAR performance on the surface data input

The sensitivity of STAR performance to the size of input surface data was evaluated through three additional tests: two using smaller rectangular surfROIs and one based on three selected spatial points. The two smaller surfROI (ROI1 and ROI2) were obtained by symmetrically shrinking the sides of the original surfROI (ROI0) by 20% and 50% respectively. The three points were selected within the ROI0 that have largest temporal variation, while ensuring that each pair of points was spatially separated by at least half of the original surfROI dimension. In the test with three surface points as input, a multi-layer perceptron was implemented as the Surf2DefNet.

**Figure 9.**
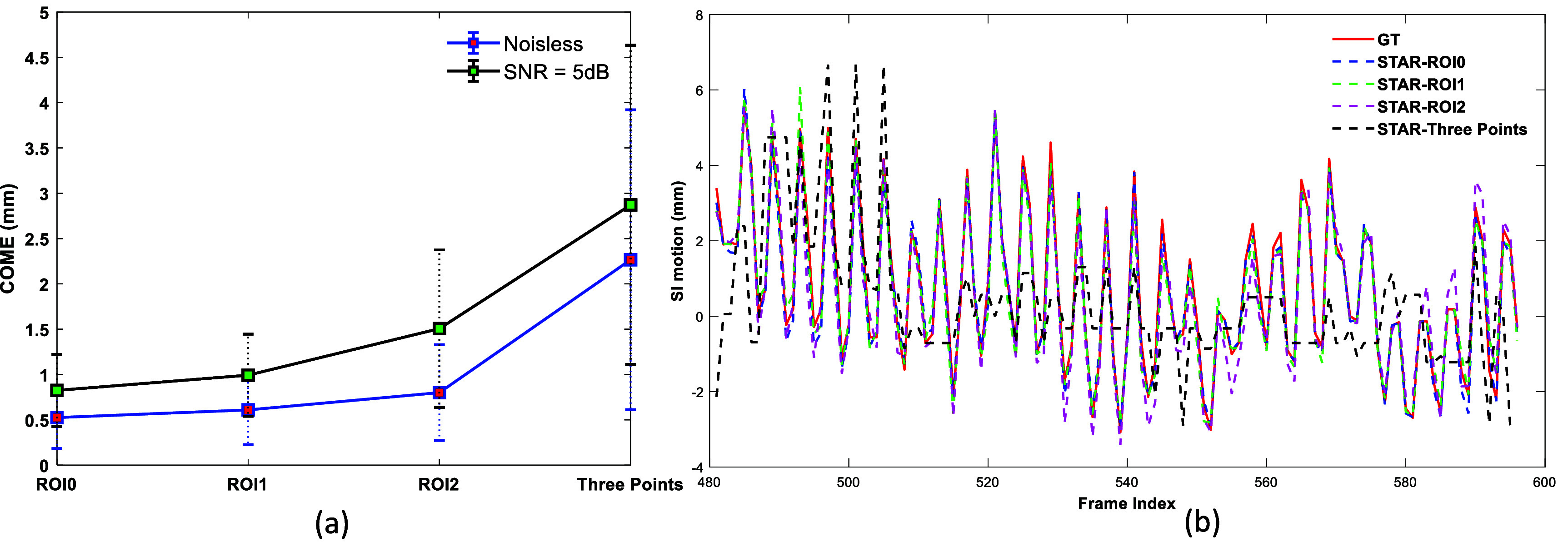
(a) Error bar plot of the mean COME for all ten patients. (b) SI motion profile comparisons for STAR against the ‘ground truth’ (GT) for one representative patient.

Figure 9(a) shows the error bar plot of the mean COME for all ten patients. The localization accuracy slightly decreases when smaller rectangle surfROIs are used, thought it remains within 1 mm with noiseless data. In contrast, when using three-point input, the positional deviation becomes more pronounced, exceeding 2 mm. Figure 9(b) presents the SI motion profile comparisons of STAR with different surface inputs against the ‘GT’ for one selected patient (P4), demonstrating larger positional deviations when smaller surfROI and three-point inputs are used.

## 4. Discussion

In this study, we developed a novel imaging technique, STAR imaging, for intra-treatment image guidance without additional ionization radiation. The STAR imaging is building on two machine-learning based algorithms: 1. A PMF-STINR technique that reconstructs a dynamic CBCT sequence (one CBCT for each x-ray projection) and solves a corresponding motion model from a conventional pretreatment CBCT scan [36], and 2. A CNN-based, Surf2DefNet, which is trained based on dynamic CBCT images and the motion model from PMF-STINR, to directly predict internal 3D anatomical deformation from intra-treatment surface images to infer real-time CBCTs.

We evaluated STAR imaging on both digital phantom (XCAT) and patient data using simulated surface images derived from boundary voxels under noise-free and noisy conditions. For the phantom with a regular (*X*1) and an irregular (*X*2) motion profile, and all patients (P1–P10), the mean tracking error measured by COME was below 1 mm in all cases except P4 (1.3 mm). These results, along with high PCCs (>0.9), demonstrated excellent motion tracking accuracy. DICE remained consistently high (⩾0.89), and the RE metric was low, indicating strong spatial fidelity of the reconstructed STAR CBCT images. While P4 exhibited a slightly higher deviation compared with other patients, the COME (1.3 mm) and HD95 (2.78 mm) remained within one and two pixels, respectively. Note that slightly larger values in the quantitative metrics were observed in the phantom study when comparing the STAR images to the phantom ‘GT’. It is due to the uncertainty passed along from PMF-STINR, which is used to generate the motion model and its reference CBCT to drive STAR imaging. Although patient cohorts and evaluation methods differ, the mean localization error of 0.48 mm (0.78 mm for noisy surface image at SNR = 5 dB) in the patient study compares favorably to the 1.75 mm error reported by Huang *et al* [30] and 1.64 mm by Wei *et al* [32].

**Figure 10.**
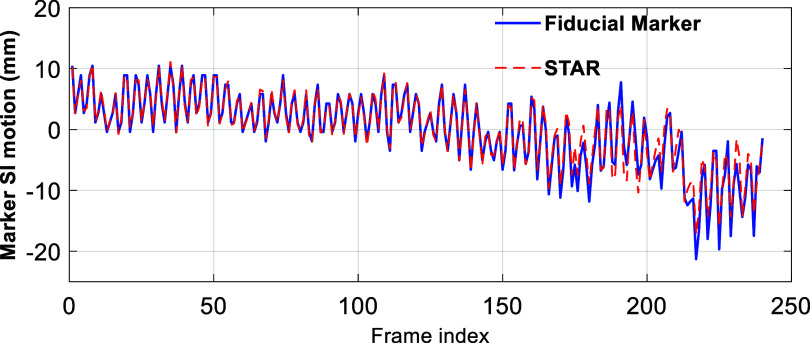
Comparison of SI motion between marker positions on acquired CBCT projections and those projected from corresponding STAR images.

Note the 3D tumor localization accuracy of STAR was evaluated using dynamic CBCT images as ‘GT’. To further validate STAR’s performance, we performed an additional assessment on one dataset (P3) in which the implanted fiducial marker was clearly visible on the cone-beam projections and served as a reference. Figure 10 demonstrated the motion trajectory comparison in SI direction between the marker positions identified on the acquired CBCT projections and the cone-beam-projected marker positions derived from the corresponding STAR images. The SI positional deviation computed on the imager plane was 2.25 ± 1.44 mm, demonstrating the motion tracking accuracy of the STAR imaging.

STAR imaging is built on a Surf2Def network trained using a dynamic CBCT set reconstructed by PMF-STINR [36], a precursory network designed to capture dynamic motion from a patient-specific, pre-treatment CBCT scan. The PMF-STINR framework reconstructs a CBCT for each cone-beam projection to capture the dynamic volumetric motion, by using a low-rank, B-spline-based motion representation to tackle the ill-posed spatiotemporal reconstruction problem. Many previous studies have found that B-spline-based representation can capture the anatomical motion and deformation accurately [40]. Though voxel-based motion representation approaches, such as optical flows [41, 42], can better represent fine-detailed motion, they have substantially more parameters to solve and are difficult to implement in the current PMF-STINR framework for the underdetermined system (limited x-ray projections available for reconstruction). Our recent study on Gaussian representation [43] has found that it can better capture the sliding motion than B-splines, by allowing more representation flexibility. Future studies are warranted to investigate potential strategies to further enhance STAR’s capabilities to represent the motion details.

Compared to existing methods based on planning 4D CTs [30–35], building motion models based on dynamic CBCTs reconstructed just before the treatment delivery offers several unique advantages:

(a) *Consistency of motion eigen-modes*. Due to anatomical changes between the time of acquiring simulation 4D CTs and the treatment delivery (often days/weeks after the simulation), the motion eigen-modes ${e_i}$ obtained from 4D CTs can be inconsistent with the motion modes during treatment, which results in wrong motion characterization and image reconstruction. Inter-fraction motion variations were reported in the literature [44], highlighting the importance of using an up-to-date motion model for each treatment fraction.
(b) *Faithful representation of regular/irregular motion*. Unlike conventional 4D CT or 4D CBCT, which assumes a regular, periodic motion by phase sorting, dynamic CBCT captures the full pattern/range of motion because each 3D image in the series corresponds to a single projection in data acquisition, and can capture both regular and irregular motion faithfully.
(c) *Enhanced image quality for motion modeling*. The periodic motion assumption of standard 4D CT/CBCT reconstruction also results in motion binning/sorting artifacts that degrade the image quality, in the context of irregular motion. Even for regular motion, the limited temporal resolution from a usual 10-phase binning also leads to intra-phase residual motion artifacts. By dynamic CBCT reconstruction, such challenges are fully addressed, leading to better image quality and a more accurate motion model.

Several remarks on Surf2DefNet are in order. As shown in figure 2, Surf2DefNet is a lightweight, feed-forward CNN, designed to maximize the computational efficiency for fast inference. Given the highest frame rate (25–30 Hz) of the surface images acquisition in the VisionRT system (VisionRT Ltd, London, UK) [45, 46], the overall time for STAR to generate a CBCT is less than 60 ms. Of this, less than 20 ms is spent estimating the three temporal coefficients for each Cartesian component of the MVF. Therefore, the STAR imaging system achieves a temporal resolution well below 500 ms, meeting the real-time imaging requirements specified in the AAPM task group report [47]. Since Surf2DefNet is trained on the anatomy and motion model acquired immediately before the treatment, it is not susceptible to the changing anatomy/motion pattern/motion correlation challenges experienced by existing methods. As a personalized model trained on each individual’s pretreatment dynamic CBCTs, it also avoids the generalizability issues common to deep learning models. Although Surf2DefNet was trained with simulated noiseless surface images, testing results with noisy surface inputs demonstrated the robustness of the STAR imaging system (figures 6 and 8(b), tables 1 and 2).

STAR imaging is an organic combination of PMF-STINR and Surf2DefNet. It enables real-time 3D imaging for intra-treatment image guidance for lung SBRT and can be potentially adapted to track abdominal tumors. STAR imaging can be deployed on modern radiotherapy treatment platforms equipped with both kV CBCT and optical imaging systems, which are widely available in many academic/community cancer centers. In addition to intra-treatment tumor motion management, the real-time CBCT images from STAR provide a basis for reconstructing the delivered dose, which supports outcome modeling, prediction, and adaptive treatment delivery. Integrating STAR into clinical workflow would involve the following steps:

(1) performing a standard CBCT scan for initial patient setup,
(2) reconstructing a PMF-STINR-based dynamic CBCT from the standard CBCT acquisition,
(3) one-the-fly training of Surf2DefNet, and
(4) acquiring real-time surface images for STAR image inference/generation.

However, several developments are needed before clinical translation. First, it took about three hours to reconstruct 2000 frames of dynamic CBCT via PMF-STINR on a NVIDIA V100 card [36]. We plan to utilize a warm-start training strategy to leverage the planning 4D CT or the dynamic CBCTs from a previous treatment fraction to reduce the image reconstruction time to under five minutes. Many strategies, such as online-offline combination [48], meta-learning [49], and our recent developed prior-adaptive [50] and Gaussian representation strategies for faster dynamic CBCT reconstruction [43] will be explored to achieve this goal. Specifically, our strategy will use a daisy chain-based scheme that adaptively fine-tunes the dynamic CBCT model based on models derived from preceding treatment fractions (for fraction one, it uses a ‘virtual’ model derived from the simulation 4DCT). Second, although STAR images are free of motion artifacts, we observed image blurring in both phantom and patient studies, as well as shading and streaking artifacts in patient images. These are inherited from the PMF-STINR-based dynamic CBCT reconstruction, and further improvements of STAR image quality can be achieved by applying additional preprocessing steps to reduce these artifacts, as discussed in detail in [36]. Third, Surf2DefNet can be further optimized to enhance imaging performance. In the current study, a rectangular-shaped surfROI was manually selected for each patient as the input to the Surf2DefNet. As shown in figure 9, this preliminary study demonstrated that tumor localization accuracy depended on the size of input surface data. For a rectangular surfROI input, the accuracy remained within 1 mm with a 50% reduction in surfROI size (ROI2). However, the positional error was more evident when using three-point input. In the future, the surfROI selection will be automated and optimized for both efficiency and accuracy. Finally, studies have shown baseline shifts of internal motion may cause accumulation of tracking error [51, 52]. Since STAR uses a surfROI area (2.5D information) rather than a single or a few surface markers to represent external motion, it is expected to be more robust against the internal-external motion correlation changes than conventional surface marker-based models. Motion augmentations that simulate large amplitude/frequency variations and baseline changes can also be added to Surf2Def training to yield more robust STAR models. In case of substantial correlation changes between surface signals and internal motion that warrant model updates, acquiring intermediate x-rays during the treatment for periodical model validation and potential recalibration of the surface-internal correlation may also alleviate the problem.

Our current work focused on estimating the real-time 3D images at the current state. For clinical utility in motion management systems (e.g. MLC tracking), it is often necessary to predict the tumor’s future position to compensate for system latencies. For a short-term prediction, a linear extrapolation may provide a reasonable estimation, however a prediction for a longer time period may need a separate, more advanced learning algorithm such as the long-short term memory strategy (LSTM). For MLC tracking purposes, a short-time prediction may suffice. The comparison between short-term and long-term predictions for real-time adaptive radiotherapy is warranted in future investigations.

There are some limitations of the current study. First, the surface images were simulated using threshold-based edge detection on the reconstructed dynamic CBCT images, which may differ from the acquired surface images by the optical camera systems. We will conduct physical phantom experiments in the future to characterize this difference for potential corrections. Second, we only evaluated the STAR imaging on ten patients. In the future, we plan to perform a comprehensive patient study to include patients treated with lung SBRT in our institute and other centers to evaluate the robustness of this imaging technique. Third, we did not include a comparative evaluation of STAR imaging with other 4D CT/CBCT-based motion modeling approaches for intra-treatment tumor tracking in this study. Such a comparison will be undertaken in future work.

## 5. Conclusion

We demonstrated the feasibility and performance of STAR imaging as a noninvasive solution for intra-treatment real-time 3D image guidance. Without incurring additional ionizing radiation, STAR achieved <2 mm target tracking accuracy, along with high DICE and PCCs in both phantom and patient studies under both ideal and noisy surface image input. The robustness and high fidelity of the reconstructed images make STAR imaging a promising approach for intra-treatment motion management, offering accurate 3D visualization to ensure precise tumor targeting and to support advanced adaptive treatment techniques.

## Data Availability

The data cannot be made publicly available upon publication because they contain sensitive personal information. The data that support the findings of this study are available upon reasonable request from the authors.

## Appendix

The projection data acquisition and the CBCT image reconstruction parameters are listed in table 3.

**Table 3.** CBCT projection acquisition and image reconstruction parameters for patients.

Patient ID	Source^a^	Scan mode	Projection size^b^	Pixel size (mm^2^)	kVp/mA/mS	SAD^c^ (mm)/SDD (mm)	Reconstructed CBCT voxels	Voxel size (mm^3^)
P1	MDACC	FF	512 × 384 × 1983	0.776 × 0.776	120/80/25	1000/1500	200 × 200 × 100	2 × 2 × 2
P2	UTSW	FF	1024 × 768 × 500	0.388 × 0.388	80/20/10	1000/1500	200 × 200 × 100	2 × 2 × 2
P3	SPARE	HF	1006 × 750 × 2416	0.388 × 0.388	120/20/20	1000/1500	300 × 300 × 102	2 × 2 × 2
P4	SPARE	HF	1006 × 750 × 2918	0.388 × 0.388	120/20/20	1000/1500	300 × 300 × 102	2 × 2 × 2
P5	UTSW	HF	1024 × 768 × 895	0.388 × 0.388	125/15/20	1000/1500	310 × 310 × 102	2 × 2 × 2
P6	UTSW	HF	1024 × 768 × 895	0.388 × 0.388	125/15/20	1000/1500	300 × 300 × 102	2 × 2 × 2
P7	UTSW	HF	1024 × 768 × 894	0.388 × 0.388	125/60/20	1000/1500	300 × 300 × 102	2 × 2 × 2
P8	UTSW	HF	1024 × 768 × 894	0.388 × 0.388	125/15/20	1000/1500	300 × 300 × 102	2 × 2 × 2
P9	UTSW	HF	1024 × 768 × 894	0.388 × 0.388	125/15/20	1000/1500	300 × 300 × 102	2 × 2 × 2
P10	UTSW	HF	1024 × 768 × 894	0.388 × 0.388	125/15/20	1000/1500	300 × 300 × 102	2 × 2 × 2

^a^ MDACC: MD Anderson Cancer Center [53]. SPARE: SPARE Challenge [39]. UTSW: University of Texas Southwestern Medical Center.

^b^ Width (in pixel number) × height (in pixel number) × *N_p_* (number of projections).

^c^ SAD: source-to-axis distance. SDD: source-to-detector distance.
